# Label-free differentiation of human pancreatic cancer, pancreatitis, and normal pancreatic tissue by molecular spectroscopy

**DOI:** 10.1117/1.JBO.27.7.075001

**Published:** 2022-07-25

**Authors:** Christian Teske, Christoph Kahlert, Thilo Welsch, Katja Liedel, Jürgen Weitz, Ortrud Uckermann, Gerald Steiner

**Affiliations:** a University Hospital Carl Gustav Carus, Technische Universität Dresden, Department of Visceral, Thoracic and Vascular Surgery, Dresden, Germany; b National Center for Tumor Diseases (NCT/UCC), Dresden, Germany; c University Hospital Carl Gustav Carus, Department of Neurosurgery, Dresden, Germany; d Technische Universität Dresden, Department of Anaesthesiology and Critical Care Medicine, Clinical Sensoring and Monitoring, Faculty of Medicine, Dresden, Germany

**Keywords:** infrared spectroscopic imaging, supervised classification

## Abstract

**Significance:**

Pancreatic ductal adenocarcinoma (PDAC) is one of the leading causes of cancer deaths with a best median survival of only 40 to 50 months for localized disease despite multimodal treatment. The standard tissue differentiation method continues to be pathology with histological staining analysis. Microscopic discrimination between inflammatory pancreatitis and malignancies is demanding.

**Aim:**

We aim to accurately distinguish native pancreatic tissue using infrared (IR) spectroscopy in a fast and label-free manner.

**Approach:**

Twenty cryopreserved human pancreatic tissue samples were collected from surgical resections. In total, more than 980,000 IR spectra were collected and analyzed using a MATLAB package. For differentiation of PDAC, pancreatitis, and normal tissue, a three-class training set for supervised classification was created with 25,000 spectra and the principal component analysis (PCA) score values for each cohort. Cross-validation was performed using the leave-one-out method. Validation of the algorithm was accomplished with 13 independent test samples.

**Results:**

Reclassification of the training set and the independent test samples revealed an overall accuracy of more than 90% using a discrimination algorithm.

**Conclusion:**

IR spectroscopy in combination with PCA and supervised classification is an efficient analytical method to reliably distinguish between benign and malignant pancreatic tissues. It opens up a wide research field for oncological and surgical applications.

## 1 Introduction

**Fig. 1:**
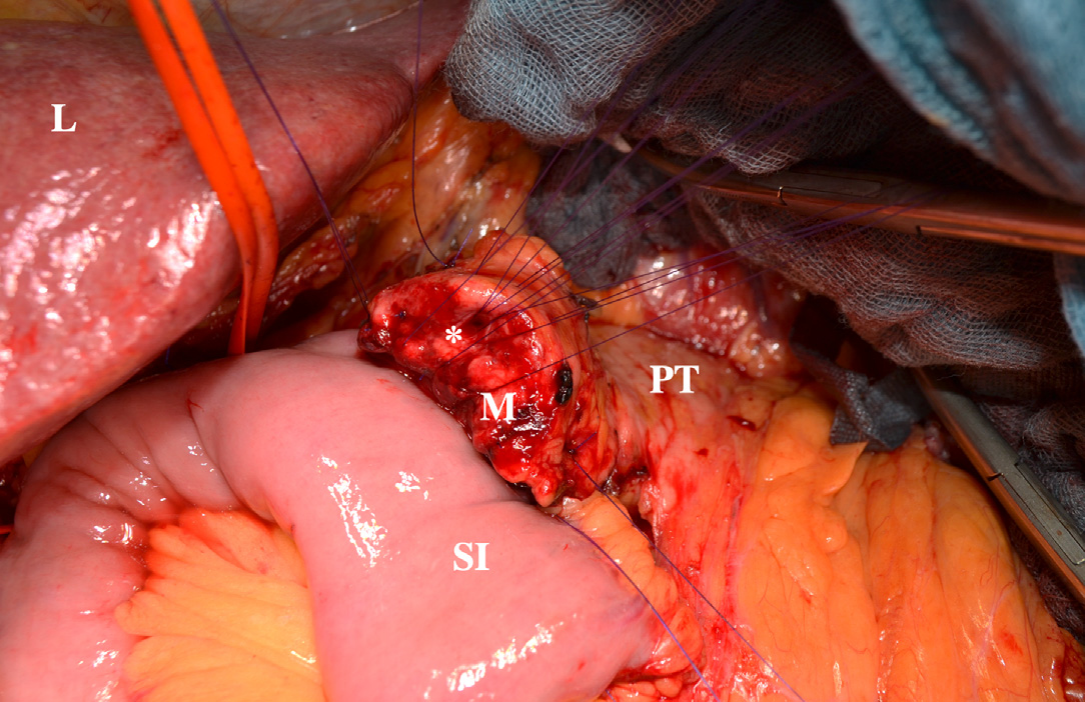
Intraoperative image of a pancreatic head resection for PDAC. The image displays the situs after having resected the tumor. The pancreatic tail (PT) leads to the resection margin (M) with the pancreatic duct (*) in the center. The liver (L) and the small intestine (SI) are located closely.

Pancreatic ductal adenocarcinoma (PDAC) is the second leading cause of cancer-related deaths involving the digestive system after colorectal cancer among men and women in western countries.^1^ Modern multimodal treatment plans including chemotherapy and surgical resection still do not improve overall survival beyond 4.5 years.^2^^,^^3^ Curative pancreatic surgery for malignant neoplasms is highly demanding, with optimal results achieved when microscopically tumor-free resection margins are obtained. Therefore, differentiation of normal pancreatic tissue, inflammatory pancreatitis and PDAC is crucial. Reliable macroscopic tissue distinction is not possible for surgeons. This is especially true in the case of infiltrating tumor margins due to cellular spread into normal pancreatic tissue (Fig. 1).

However, in most cases microscopic resection margin clearance of pancreatic cancer determines the patient’s survival rate.^3^^–^^5^ Currently, the gold standard for tissue discrimination of pancreatic lesions is microscopic histopathological analysis of pre-stained samples. Frozen sections are sent to pathology intraoperatively to be microscopically analyzed for remaining tumor cells. This method is used to ensure tumor-free resection margins.

Several methods such as imaging mass spectroscopy and tumor-specific agents for fluorescence imaging have been investigated to accurately identify PDAC tissue.^6^^,^^7^ Each method slightly increases the ability to discriminate between neoplasms, inflammatory tissue and adjacent normal tissue. However, the lack of molecular information and time-consuming measurements are great limitations of these methods. A straightforward approach for the identification of pancreatic tumors is biochemical analysis on the molecular level performed by vibrational spectroscopy. Recent reports have been largely dominated by studies focusing on this method to characterize brain tumors and distinguish between glioblastoma and normal brain cells even under in situ conditions or intraoperatively.^8^^–^^13^ In particular, Fourier transform infrared (FT-IR) spectroscopic imaging is capable of showing changes in carcinogenesis-related vibrational modes in several human cancers, especially in larger areas or homogeneously structured tissue.^14^^–^^16^ However, the evaluation of IR spectroscopic data captured from biological tissue or cells is often difficult due to the extensive amount of information.^8^ Therefore, IR spectroscopic imaging is often used in conjunction with multivariate methods such as principal component analysis (PCA) to compress data and maximize and highlight small area variations.

Considering the devastating mortality rate of pancreatic cancer, there is a comprehensive clinical need for fast and reliable differentiation of pancreatic tissue. IR spectroscopic imaging has already been used for the characterization and differentiation of human PDAC and pancreatic neuroendocrine tumors.^17^ In this study a pair-wise PCA was carried out to discriminate PDAC, pancreatic neuroendocrine tumor, dysplastic pancreatic, and normal tissue by FT-IR imaging spectroscopy. The authors found that the four histologic entities can be differentiated based on their IR spectra. The present study aimed to distinguish between normal pancreatic tissue, pancreatitis, and PDAC, using IR spectroscopic imaging in combination with PCA and supervised classification. IR spectroscopic imaging was performed to identify even small tumor clusters within the snap frozen tissues and to subsequently compare the spectral profiles in structural comparison to standard histological images.

## 2 Material and Methods

### 2.1 Sample Preparation

Pancreatic tissue samples were collected from medically indicated pancreatic surgical resections at the Department of Visceral, Thoracic, and Vascular Surgery of the University Hospital Carl Gustav Carus, Technische Universität Dresden. Written informed consent was obtained from all patients prior to surgery. The study was approved by the local ethics committee (EK179052018).

Representative parts of the resected specimens were snap frozen with liquid nitrogen. For IR spectroscopy, 18-μm thick samples were placed on ${\mathrm{CaF}}_{2}$ slides using a cryotome. Serial section samples were mounted on standard slides and stained according to regular hematoxylin and eosin (HE) staining protocols. HE-stained samples served as histological control for tissue classification and area recognition. In total, 20 tissue samples were analyzed from an existing tumor bank according to the described protocol: seven normal pancreas tissues, eight PDAC samples, and five pancreatitis specimens. The samples were obtained from eight patients with PDAC (four male and four female, median age 72 years) and from five patients with chronic pancreatitis (four male and one female, median age 49 years).

### 2.2 IR Spectroscopy

IR spectra were acquired in transmission mode with an FT-IR Tensor 27 spectrometer equipped with a Hyperion 3000 IR microscope (both from Bruker Optics GmbH, Ettlingen, Germany). A 15× Cassegrain objective (0.4 NA) imaged an area of $175\times 175\;{\mu m}^{2}$. The radiation was collected by a 64×64 mercury cadmium telluride focal plane array detector. Composite images of several square millimeters depending on the sample size and morphology (max. 12×4 individual images, 2.1  mm×0.7  mm) were captured in an automated stepwise manner by moving the sample stage. All individual IR spectroscopic images were recorded with 2×2 binning, so the dimension was reduced to 32×32=1024 spectra. Each composite image comprises 4×12×1024=128×384=49,152 spectra. Before measuring each sample, a background image was recorded from the pure ${\mathrm{CaF}}_{2}$ slide. The spectral resolution was set to $6\;{\mathrm{cm}}^{-1}$ to improve the signal-to-noise ratio, reduce the size of the spectral data set, and ensure that all prominent bands appear clearly in the spectrum, including those with medium intensity. For each pixel, 100 interferograms were collected, co-added, and Fourier transformed by applying Blackman–Harris apodization and a zero filling factor of 1. The sample-to-background spectrum ratio and transmission spectra were converted to absorbance values. An atmospheric compensation was performed to subtract contributions of residual water vapor bands from the spectra using the routine in the OPUS software package (Bruker Optics GmbH, Ettlingen, Germany). Finally, spectral data sets were reduced to the spectral region of 950 to $1800\;{\mathrm{cm}}^{-1}$ and stored for subsequent data analysis.

### 2.3 Data Processing and Evaluation

Data processing and analysis were performed using MATLAB packages (version 7, MathWorks, Inc., Natick, Massachusetts). First, raw data preprocessing procedures were carried out involving the removal of outliers, a linear baseline correction, and a normalization of each absorbance value of a spectrum to the integral absorbance. Outliers are spectra that are obviously not associated with tissue or spectra with a maximum absorbance value larger than 2 or smaller than 0.05. The baseline of each spectrum was corrected using the *msbackadj* function in the MATLAB Statistics Toolbox. Afterward, the spectra were area normalized to eliminate differences due to sample thickness or variations in tissue density. The *eig* function in the basic MATLAB package was used for PCA calculations. A k-means cluster analysis was performed on area normalized spectra in MATLAB using the k-means function with the distance parameter Mahalanobis. In according to the elbow criteria, the estimated number of clusters was found to be between 7 and 8. Therefore, the number was set to 10.^18^ K-means cluster analysis was performed to identify areas of tumor tissue and normal pancreatic tissue within the tissue sample. Spectra that could be clearly assigned to tumor, pancreatitis, or normal tissue were used to build up the training set for the PCA-based supervised classification of unknown tissue samples.

### 2.4 Classification

A three-class classification model for normal tissue, tumor tissue, and pancreatitis was developed based on the final pathology report in combination with the calculated PCA score values. PCA was performed to reduce the large amount of data contained in the measured spectra into a few important PCs for identification PDAC, pancreatitis, and normal tissue. PC scores were used as input for linear discriminant analysis using a leave-one-out strategy on the training set. Classification was performed using the *classify* function in the MATLAB Statistics Toolbox together with a leave-one-out cross-validation to establish the calibration model. The training set was built by random selection of 25,000 spectra for each class and score values of the first 30 PCs resulting in a training data set of three classes ×25,000 spectra ×30 score values. The first 30 PCs achieved the maximum overall accuracy and represented 99.6% of the data set’s variance (Fig. S1 in the Supplemental Material). Using this approach, each spectrum is represented by 30 values rather than 441 data points.

For classification of the independent test samples, PCA was performed for all spectra individually. Subsequently, all spectra were classified stepwise based on the score values of the first 30 PCs. The algorithm provided the probability of membership to normal, tumor, or pancreatitis tissue classes for both the training and test set. The probability values were converted into an RGB image.

## 3 Results and Discussion

**Fig. 2:**
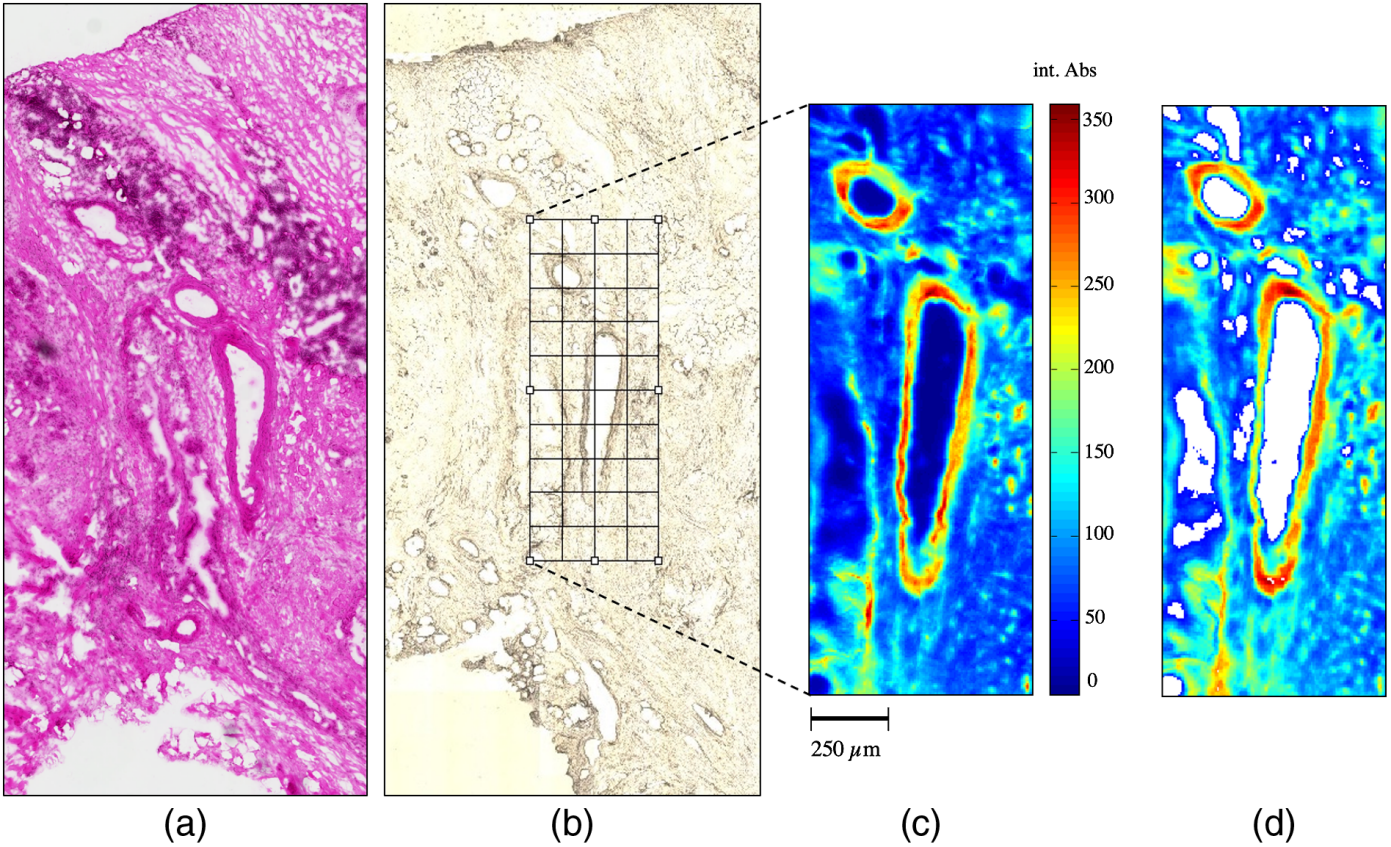
(a) Histopathologic HE-stained image served as area recognition and standard tissue classification. (b) Microscopic transmission image of a cryo-preserved specimen of representative tissue section with an overlaid grid of the area mapped by IR spectroscopic imaging. (c) IR spectroscopic raw data bright field image (integrated absorbance [int. Abs.] between 950 and $1800\;{\mathrm{cm}}^{-1}$) with bar representing width of the image. (d) Image of selected preprocessed spectra from (c). Spectra of white pixels were identified as outliers and removed from the data set.

Tumor tissue samples were captured and analyzed in a visible transmission mode and an IR spectroscopic image (Fig. 2). HE-stained standard histopathological specimens served as control. The grid overlay [Fig. 2(b)] corresponds to the mapping matrix of the IR spectroscopic images. The sample was imaged by 4×12 individual spectroscopic images containing a total of 4×12×1024=49,152 spectra. Binning reduces the spatial resolution and storage volume of the data by a factor of four, but increases the signal-to-noise ratio and significance of the spectral profile. Spectra obviously not associated with tissue or areas with dominant artifacts including a strong background signal were classified as outliers and removed from the data set. The image calculated from preprocessed spectra is represented in Fig. 2(d).

**Fig. 3:**
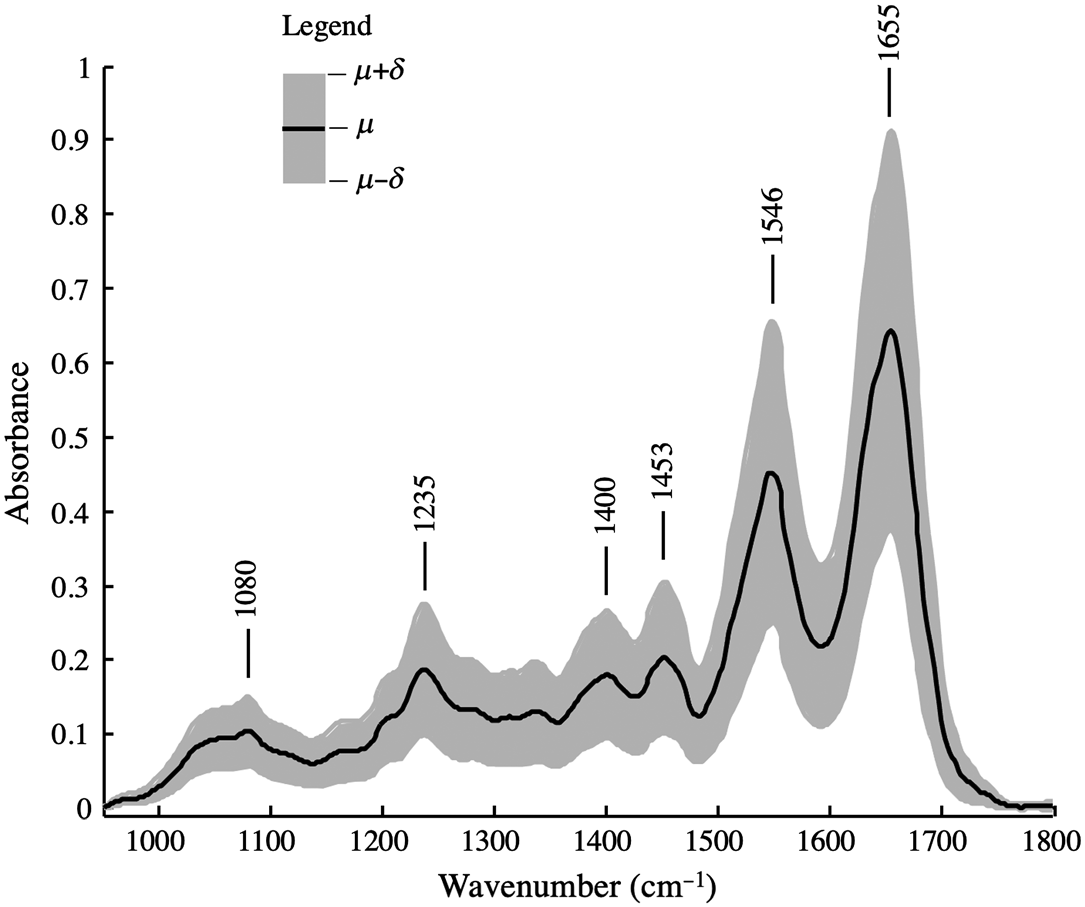
Mean spectrum (μ, black curve) and standard deviation area (δ, gray band) of the sample represented in Fig. 2. Mean spectrum was calculated from the preprocessed data set of the image shown in Fig. 2(d).

In the first step, the mean spectrum and standard deviations of the data set shown in Fig. 2 were calculated and applied (Fig. 3). The spectrum is dominated by the amide I band at $1650\;{\mathrm{cm}}^{-1}$ (C═O stretching) and amide II band at $1550\;{\mathrm{cm}}^{-1}$ (N-H bending) of the amide groups comprising the peptide linkages of proteins (Table 1).

**Table 1:** Assignment of vibration modes. The symbols refer: ν, stretching mode; δ, bending mode; s, symmetric, as antisymmetric.

Position (${\mathrm{cm}}^{-1}$)	Assignment
1025	ν(C─O), carbohydrates, glycogen
1080	$\nu_{s}({\mathrm{PO}}_{2^{-}})$ phosphate groups of nucleic acids, phosphodiesters
1200, 1208	Collagen, $\nu_{\mathrm{as}}({\mathrm{PO}}_{4^{-}})$
1235	Amide III
1240	$\nu_{\mathrm{as}}({\mathrm{PO}}_{2^{-}})$ phosphate groups of nucleic acids
1340, 1346	$\delta ({\mathrm{CH}}_{2})$, collagen
1396	$\delta ({\mathrm{CH}}_{3})$, methyl groups (mainly protein side chains)
1400	$\delta ({\mathrm{CH}}_{3})$, methyl groups (mainly protein side chains)
1448	$\delta_{\mathrm{as}}({\mathrm{CH}}_{3})$, methyl groups
1453	$\delta_{\mathrm{as}}({\mathrm{CH}}_{3})$, methyl groups
1516	Amide II, C─H bending vibration modes (from rings)
1522	ν(C═N), ν(C═C)
1546	Amide II
1560	Amide II
1590	Ring C─C stretch
1618	Ring C─C stretch, collagen
1650, 1655	Amide I, α-helix
1675	Amide I, turns
1715	ν(C═O)

Two more significant bands appear around 1453 and $1400\;{\mathrm{cm}}^{-1}$, which were assigned to ${\mathrm{CH}}_{3}$ bending vibrations of methyl groups. The band at $1235\;{\mathrm{cm}}^{-1}$ corresponds to the amide III band and the group between 1000 and $1150\;{\mathrm{cm}}^{-1}$ is mainly composed of absorption bands of C─O and ${\mathrm{PO}}_{4^{-}}$ groups of nucleic acids, phospholipids, and carbohydrates. The most important spectral bands assigned to vibrational modes are summarized in Table 1. Of note is the standard deviation is 0.1 au. This indicates a large variance in biochemical composition, therefore the average considered in isolation can be misleading. Furthermore, the most common types of PDAC are histologically heterogeneous with different tumor tissue compartments such as tumor cell clusters, stromal desmoplastic tissue, and infiltration zones.

**Fig. 4:**
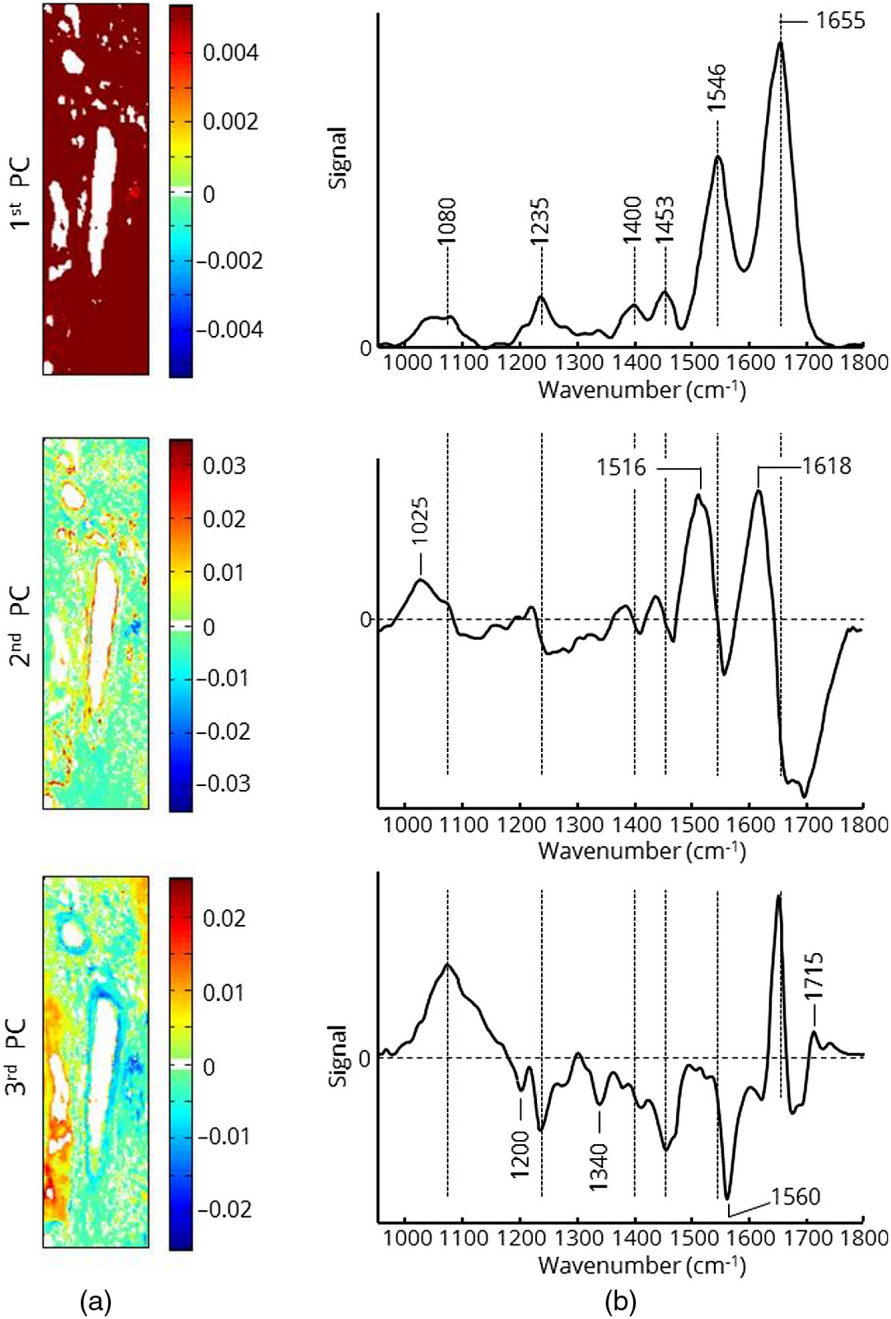
The first three PCs of the spectroscopic image from Fig. 1. (a) Maps of the score values are displayed in the centered rainbow color code. (b) Corresponding loading plots to PCs.

To inspect the biochemical variations across the sample, a more sophisticated data analysis is required to extract signals from different types of tissue and cells. The mathematical investigation of spectral signals to distinguish cancer from normal tissue or to detect tumor cells infiltrating into normal tissue was performed by PCA. It was used because it is a quick way to assess variations within the spectral data set. It does not assume any explicit statistical model underlying the variance of the original spectra. The results of the first three PCs are shown in Fig. 4.

The loading plots correspond to spectral bands with the highest variations and weight the signals in the positive and negative directions. The score map reveals the weight of the loading plot for each pixel of the image. PCA is applied to the baseline corrected and area normalized spectra. The first PC represents the mean spectrum of the sample (Fig. 3). The corresponding score map only shows a few weak variations in intensity. The loading plot of the second PC shows two positive signals located at 1516 and $1618\;{\mathrm{cm}}^{-1}$. Both signals are associated with vibrational modes of proteins and can be assigned to collagen. Another positive broad signal occurs between 1000 and $1080\;{\mathrm{cm}}^{-1}$ with a maximum located at $1025\;{\mathrm{cm}}^{-1}$, representing collagen as well. The weak signal between 1680 and $1750\;{\mathrm{cm}}^{-1}$ is composed of amide I vibrational modes and modes of C═O groups. Therefore, the second PC is interpreted to indicate collagen, which is supported by the histological image (Fig. 2). The red and orange pixels in the score map correspond to blood vessel structures. The loading plot of the third PC displays strong positive signals at 1080 and $1655\;{\mathrm{cm}}^{-1}$ assigned to phosphate groups of DNA and alpha-helical substructures of proteins, respectively. The red and orange pixels of the loading plot correspond to tumor infiltrating tissue as indicated in the histopathological image (Fig. 2). A series of negative signals represents vibrational modes of ${\mathrm{CH}}_{x}$ groups and other protein structures. In particular, the pattern of signals at 1200, 1340, and $1516\;{\mathrm{cm}}^{-1}$ points to collagen. The blue and turquoise pixels in the corresponding score map show a similar pattern to that of the red and orange pixels in the score map of the second PC. The second and the third PC are the most important for classification purposes. Higher PCs predominantly represent signals without any impact on the classification, noise, or atmospheric signals.

**Fig. 5:**
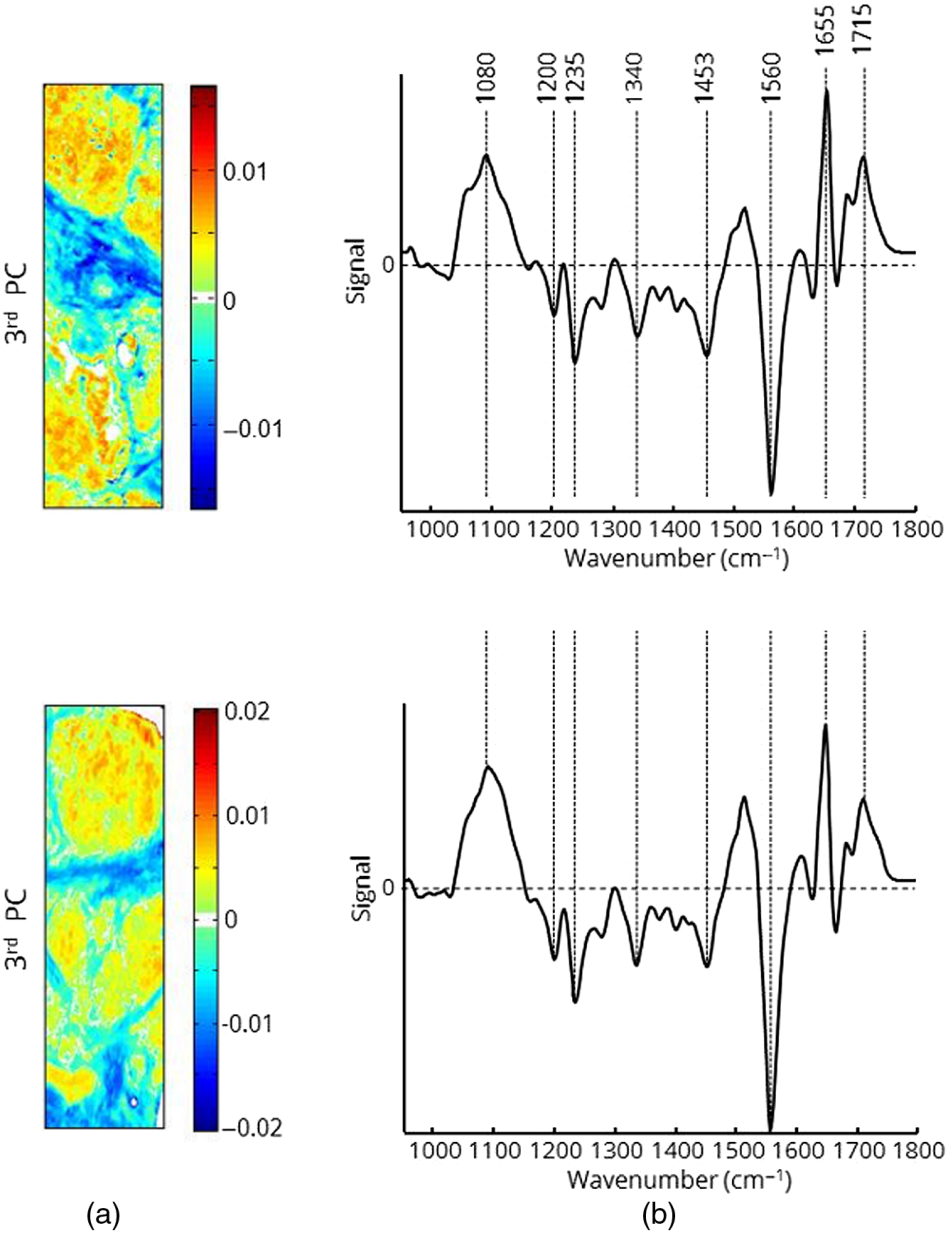
Third PC of two additional tumor samples with (a) score values and (b) loading plots. Only spectra with a score value >0.005 were selected for the training set.

The score maps show tumor-budding areas of PDAC that correspond very well with the histologic image (Fig. S2 in the Supplemental Material). Although these results suggest that PCA alone is a suitable tool to identify and highlight pancreas tumor tissue, it should be noted that a precise delineation of cancer is not always possible by PCA due to the high intrinsic variability of pancreatic tissue. Since tumor-specific spectral features may be weak and overlaid by all other common tissue signals, a supervised classification has to be performed. This requires a defined training data set before performing the classification of unknown spectra. Each class of the training set is used as a reference for the classifier. Since the quality of training data determines the accuracy of classification, it is necessary to select representative spectra for each class that cover both the general spectral feature of the class and the natural variability of these features. In particular, tumor tissue has a heterogeneous appearance with areas of different cell density and stages of development. Therefore, two more PDAC samples with histologically distinct visible tumor areas were analyzed by PCA, and score maps and loading plots calculated (Fig. 5). The corresponding histological images are represented in the supplementary part (Fig. S2 in the Supplemental Material). The spectral features of both loading plots are very similar to each other and to those of the third PC in Fig. 4. In accordance with the histological images, only spectra indicated by red or orange pixels in the third PC were selected for creation of the training set. A total of 55,000 spectra representing tumor tissue were selected.

**Fig. 6:**
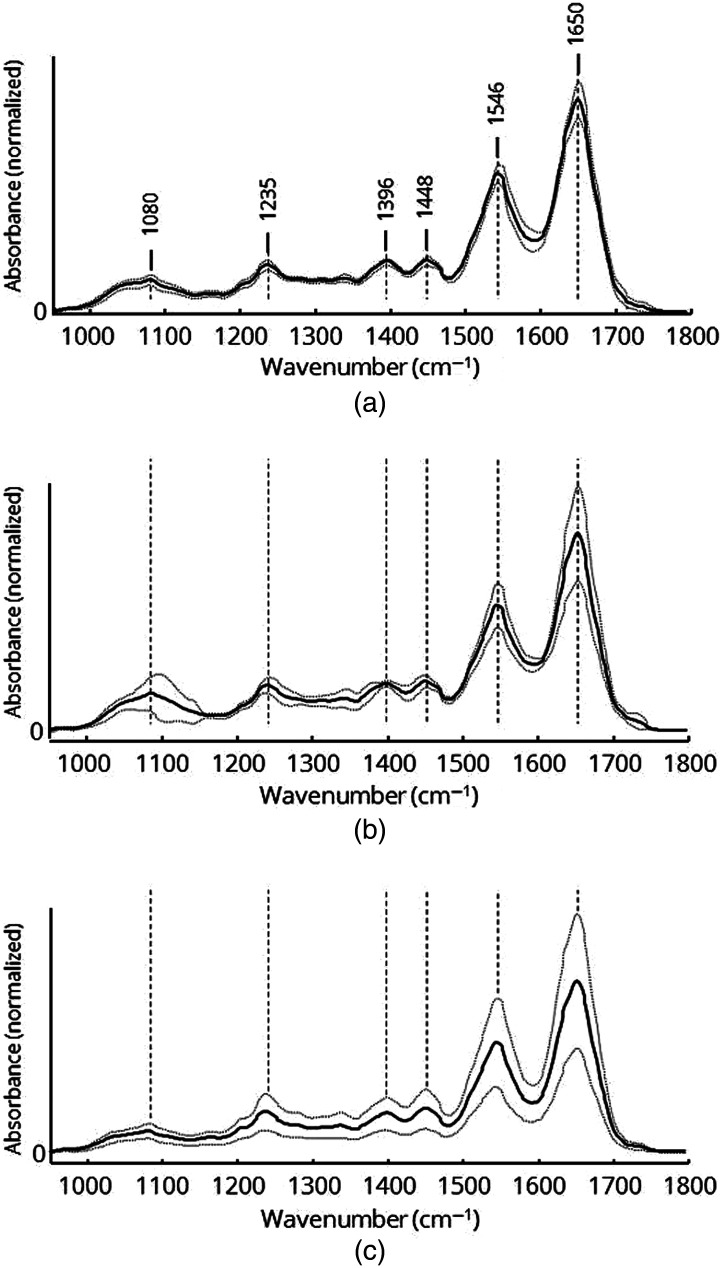
Mean spectra (solid curves) and standard deviations (dotted curves) of (a) normal tissue, (b) tumor tissue, and (c) pancreatitis tissue. Mean spectra and standard deviation were calculated from training data sets of each tissue class.

Since it is often difficult to distinguish between tumor and pancreatitis tissue, a second class of the training set was defined by spectra of pancreatitis. A third class of the training set was defined by physiological pancreatic tissue. The spectra of two representative samples of pancreatitis and normal tissue were taken for creation of the training set. Pancreatic tumor tissue is often surrounded by connective, inflammatory, and stromal tissue and associated with cellular inhomogeneity. To select only spectra of pancreatic tumor cells, a fuzzy k-means cluster analysis was performed to discriminate between tumor cells and nontumor cells. The image of the cluster assignment was compared to the histological stained micrograph of a consecutive section to identify areas of tumor cells. An example of cluster analysis and the corresponding histological image are represented in the supplementary part (Figs. S2 and S3 in the Supplemental Material). Reddish and orange pixels correspond to dense tumor clusters in the histological image. Only spectra belonging to the red and orange clusters were used as reference tumor spectra for the training set. Normal tissue and pancreatitis tissue samples were considered uniform and were not differentiated. To compare classes and find unique features, the average spectra were calculated from more than 55,000 spectra for each tissue class. The averaged spectra and standard deviations of normal, tumor, and pancreatitis tissue are shown in Fig. 6.

**Fig. 7:**
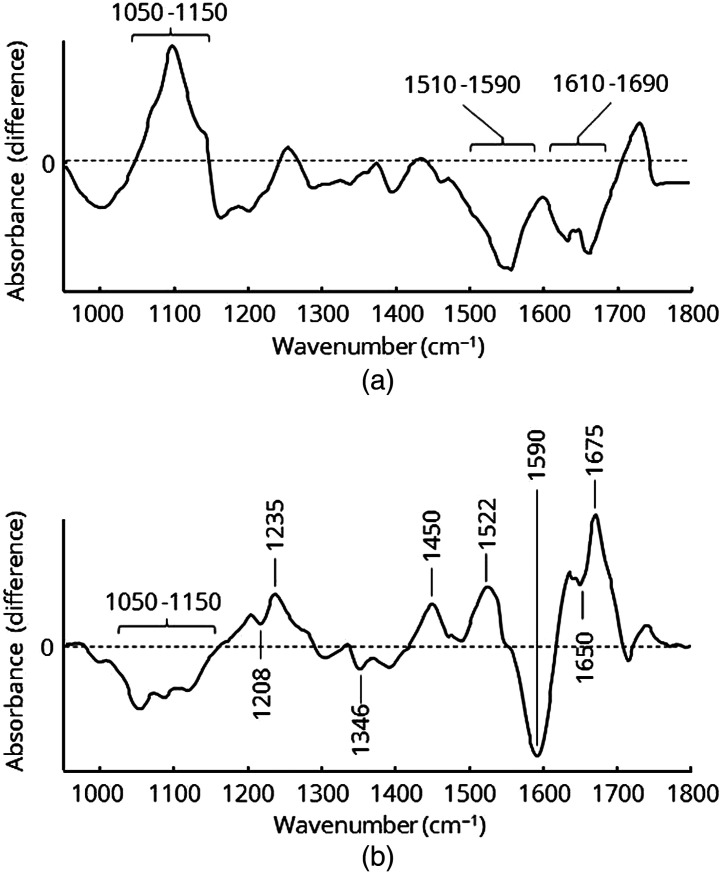
Difference spectra of the calculated mean spectra: (a) pancreatitis tissue–normal tissue, (b) tumor tissue–normal tissue.

Although the spectral profiles may appear similar, there are a few differences between the classes. While the plot of normal tissue [Fig. 6(a)] exhibits an overall low standard deviation, the spectra of tumor tissue [Fig. 6(b)] and pancreatitis tissue [Fig. 6(c)] show stronger variation, as indicated by the higher standard deviation. Another conspicuous difference is the absorption profile between 1050 and $1150\;{\mathrm{cm}}^{-1}$. Nucleic acids and carbohydrates as well as their derivatives give rise to prominent bands in this spectral region. Figure 7 highlights the spectral changes and also shows the difference spectra of tumor tissue [Fig. 7(a)] and pancreatitis [Fig. 7(b)] compared to normal tissue.

The difference spectrum between tumor and normal tissue shows three prominent features. While the spectral profile between 1050 and $1150\;{\mathrm{cm}}^{-1}$ is higher for tumor tissue compared with physiological pancreatic tissue, both the amide II (1510 to $1590\;{\mathrm{cm}}^{-1}$) and amide I bands (1610 to $1690\;{\mathrm{cm}}^{-1}$) are clearly weaker. The stronger absorption between 1050 and $1150\;{\mathrm{cm}}^{-1}$ arises mainly from the greater amount of carbohydrates due to the enhanced and altered metabolism of the tumor cells. Although the cell density in PDAC is lower compared to normal pancreatic tissue, tumor cells are characterized by a high proliferation rate. This leads to an increased amount of DNA as well as enhanced metabolic activity with a higher gene transcription rate and consequently elevated RNA levels. Hence, PDAC tissue shows a stronger absorption of nucleic acids, which also contribute to the positive difference signal. In addition, PDAC cells show altered extracellular matrix protein production, which is associated with myofibroblastic stellate cells and a remarkable density of collagen fibers.^19^^,^^20^ This desmoplastic reaction is a surrogate marker of PDAC tissue, which leads to a high fraction of connective tissue. Consequently, the intensity of the amide I and amide II bands is supposed to be higher than in normal pancreatic tissue. Due to the selection process for tumor spectra using only dense PDAC cell cluster areas, connective tumor tissue is underrepresented in the mean tumor spectrum, thereby resulting in lower amide I and amide II bands.

Pancreatitis tissue shows a lower intensity in the spectral range from 1050 to $1150\;{\mathrm{cm}}^{--1}$ compared to normal tissue but stronger signals of proteins [Fig. 7(b)]. The amide III band ($1235\;{\mathrm{cm}}^{-1}$), parts of the amide II band ($1522\;{\mathrm{cm}}^{-1}$), and also components of the amide I band ($1675\;{\mathrm{cm}}^{-1}$) are higher. The negative signal at $1590\;{\mathrm{cm}}^{-1}$ as well as the slightly lower differences at 1208, 1346, and $1650\;{\mathrm{cm}}^{-1}$ can be assigned to collagen. Pancreatitis is an inflammatory biological process within the pancreatic tissue. Different triggers (e.g., chronic alcohol consumption, biliary system alteration, and hereditary mutations) cause the migration of various cell types associated with the immune system (macrophages, lymphocytes, etc.) into the tumor tissue, where they may initiate and maintain the immune response through the secretion of mediators. Macroscopically visible variations of pancreatitis are fibrosis of the tissue and atrophy of the parenchyma. Hence, the entire biochemical profile is altered compared to normal tissue. In particular, cell atrophy and retained pancreatic secretion in combination with dilated ductal systems cause a lower density of cells, thereby resulting in weaker absorption signals between 1050 and $1150\;{\mathrm{cm}}^{-1}$. It should be noted that the explicit molecular mechanisms of pancreatitis are complex and may cause interactions within the biochemical profile which are not yet well understood and need to be examined further.

The finding of spectral differences distinguishing the class average spectra leads to the hypothesis that it might be possible to discriminate between normal, pancreatitis, and pancreatic tumor tissue. The success of a spectral classification method is dependent on the ability to factor out possible variability among the spectra within each class. In particular, supervised classification methods enable the identification of spectral patterns that are valid within a class even with intraclass variability.

**Fig. 8:**
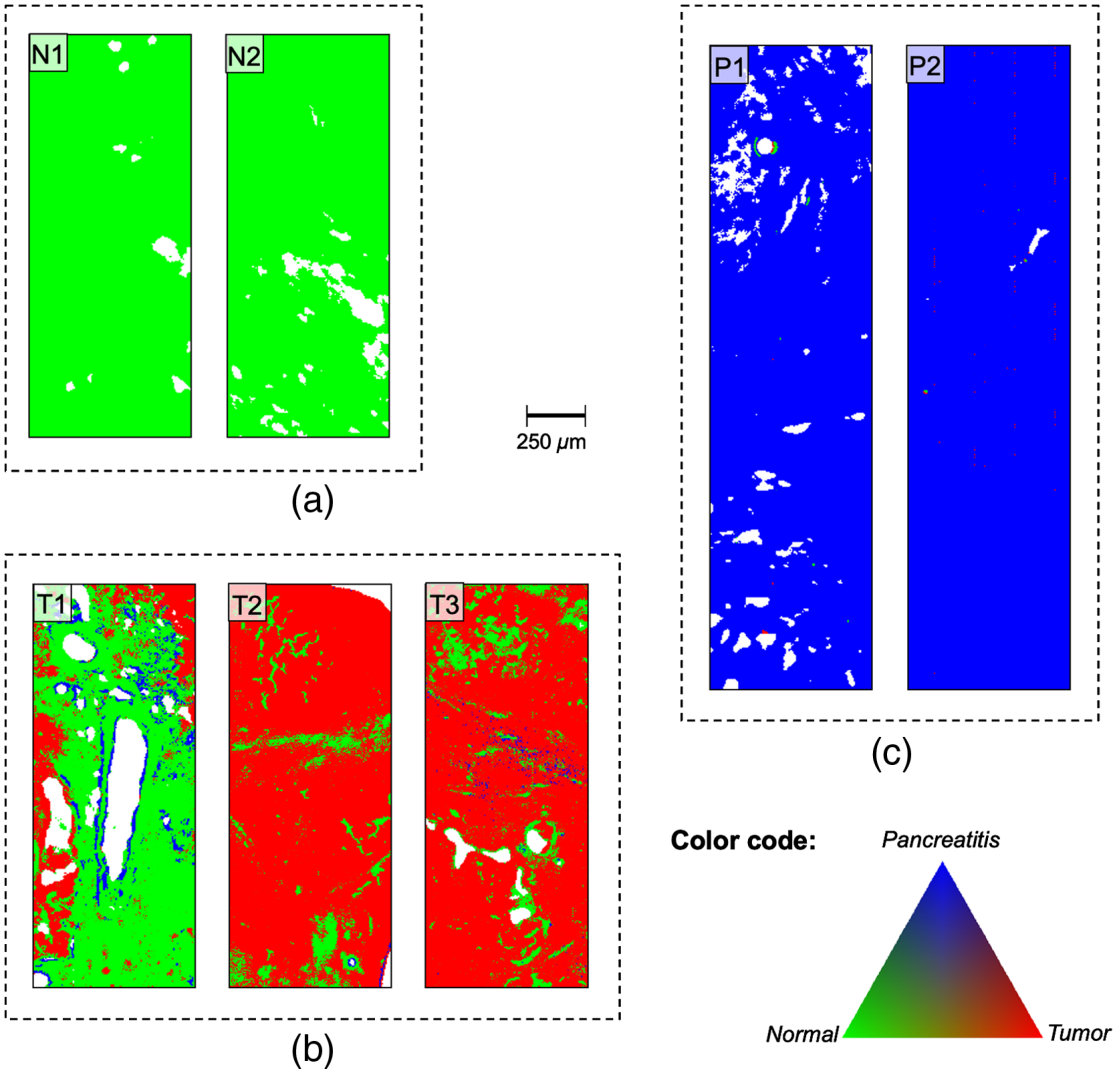
False color images of the reclassification of samples from which selected spectra were used for the training set. Reclassification of (a) normal tissue samples, (b) tumor tissue samples, and (c) pancreatitis tissue samples. N, normal tissue; P, pancreatitis; T, tumor tissue.

In the first step, 25,000 spectra of each tissue class were randomly selected for the training set. PCA was applied to the entire data set to reduce its dimension while still retaining variability. The number of PCs included in the classification model was determined based on the classification accuracy criterion. The plot of classification accuracy in relation to PC is presented in the supplementary material for all three classes (Fig. S1 in the Supplemental Material). The optimal number of PCs was defined if a global accuracy of 95% was attained. This optimal number value of reduced dimensions of PCA is reached when the score values of the first 30 PCs are used for the classification. The results of the reclassified training set are presented in Fig. 8.

Both normal (N1, N2) and pancreatitis (P1, P2) tissue samples exhibit an overall high classification accuracy. Tumor tissue sections are known to be heterogeneous and usually display infiltrating tumor cell areas but also desmoplastic stroma reaction and normal pancreatic tissue. Therefore, the classification of these samples shows quite heterogeneous RGB assignments compared to normal and pancreatitis tissues. Consequently, we have defined the term “accuracy” in the clinical context primarily to identify and to discriminate areas and clusters of tumor tissue. The tumor samples (T1 to T3) show red pixels, thus indicating tumor tissue. However, they also show green and in the case of T1 even blue pixels, corresponding to normal and pancreatitis tissues, respectively. A closer inspection of the tissue sections reveals that most of the areas classified as normal tissue within the tumor samples do not correspond to tumor cell clusters but rather to stromal tissue. Structures associated with blood vessels in sample T1 were predominantly classified as “pancreatitis.” Since the spectral selection process for the tumor category mainly used dense tumor cell cluster areas, the tumor connective tissue is underrepresented in the classifier, thus leading to misclassification in these histological areas. Physiological pancreatic features may outweigh malignant characteristics in regions of stromal tissue. The comparison of the classified samples with their histopathological HE-stained images does not show any tumor cell clusters in the normal (green) pixel areas, thus supporting this hypothesis (Fig. S2 in the Supplemental Material).

**Fig. 9:**
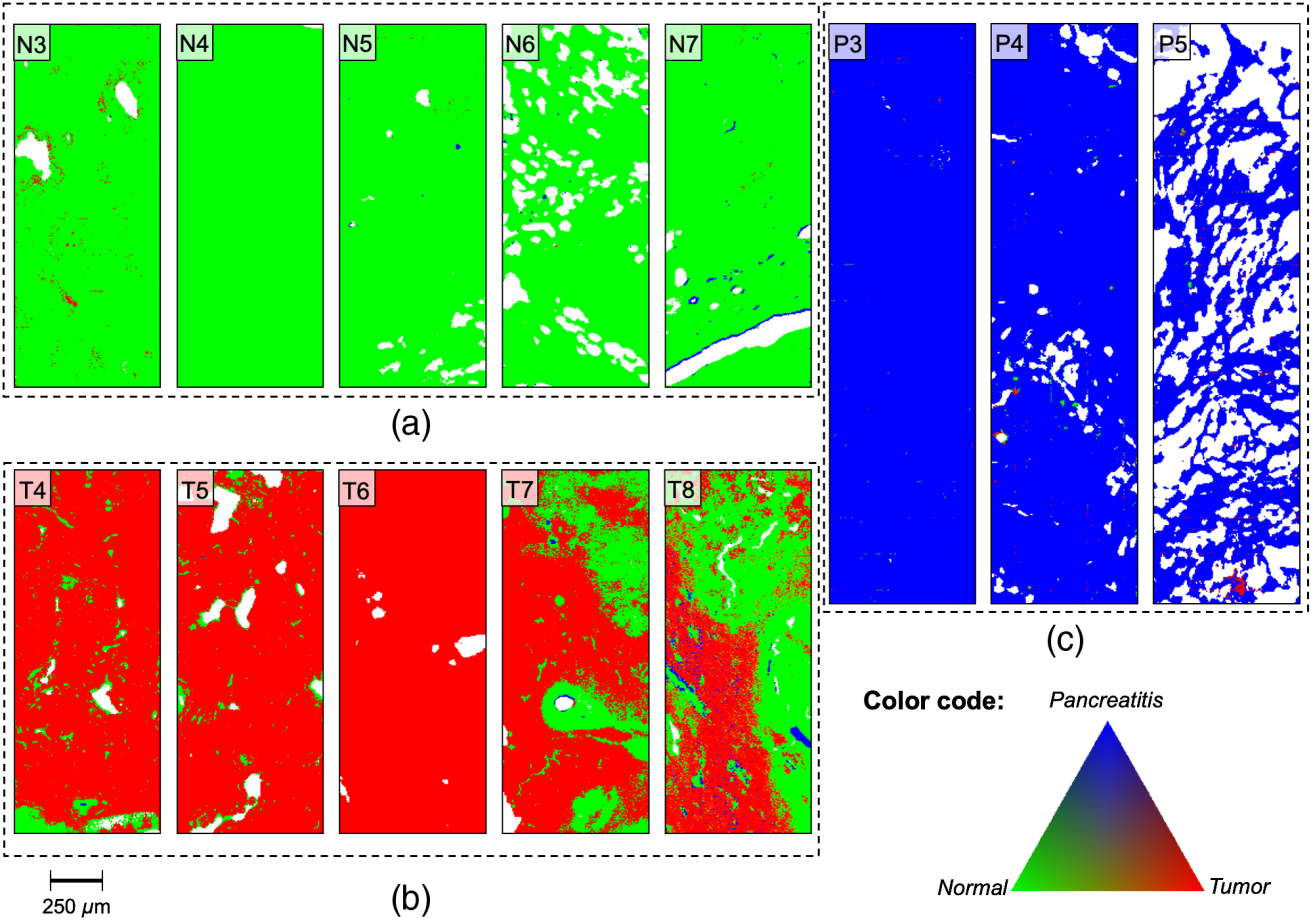
False color images of the test samples classification results. (a) Normal tissue samples, (b) tumor tissue samples, and (c) pancreatitis tissue samples. N, normal tissue; P, pancreatitis; T, tumor tissue.

The results obtained show that the classification model is successful in recognizing normal and pancreatitis tissues with an overall accuracy of close to 100% for the reclassified training set. Based on this training set and classification model, a number of “unknown” tissue samples from all three classes were analyzed (Fig. 9). Similar to the reclassification of the training set, nearly all of the spectra of normal and pancreatitis tissue samples were classified correctly. While most tumor samples were identified properly, they showed a more inhomogeneous false color image including different pixel types. Comparing the histological images shows that predominantly stromal tissue was misclassified, thus supporting the selection process hypothesis. Moreover, in the last several years pancreatic cancer research has focused on the heterogeneity of PDAC tissue. Different molecular alteration patterns have been recognized and classified into three common subtypes (exocrine-like, quasi-mesenchymal, and classical) with clinical implications and varying degrees of chemosensitivity.^21^ To date, subtype-specific spectral characterization has not been developed. With regard to the molecular pattern, there might be subtypes with normal-like spectra, which may lead to partial misrecognition due to the little number of training set samples. However, the general assignment to the class “tumor” can be defined for all five test set samples. For a further automatic classification system, a categorizing misclassification threshold would have to be evaluated based on clinical needs and safety issues. Nevertheless, IR spectroscopy is a suitable analytical method to differentiate pancreatic tissue classes in a fast and marker-free manner.

## 4 Conclusion

IR spectroscopy imaging combined with PCA and supervised classification methods have demonstrated the ability to clearly distinguish between benign and malignant pancreatic tissue in a fast and label-free way. However, further investigation of the biochemical profile of pancreatic cancer is needed to improve our understanding of tissue characteristics. Therefore, IR spectroscopy opens up a wide range of possibilities for characterizing solid human cancer entities in a markerless manner and may also lead to changes in intraoperative strategy that help achieve the best outcome for patients.
