# Supplementary material for: Label-free differentiation of human pancreatic cancer, pancreatitis, and normal pancreatic tissue by molecular spectroscopy
Source: J Biomed Opt. 2022 Jul 25;27(7):075001. doi: 10.1117/1.JBO.27.7.075001 (PMC9313287; doi:10.1117/1.JBO.27.7.075001)
Supplement: Supplementary file 1 [file JBO_027_075001_SD001.pdf]

Label-free differentiation of human pancreatic cancer, pancreatitis and normal pancreatic tissue by molecular spectroscopy

Christian Teske[1,2,*], Christoph Kahlert[1,2], Thilo Welsch[1,2,&], Katja Liedel[1,2], Jürgen Weitz[1,2], Ortrud Uckermann[3], Gerald Steiner[4]

[1] Department of Visceral, Thoracic and Vascular Surgery, University Hospital Carl Gustav Carus, Technische Universität Dresden, Germany

[2] National Center for Tumor Diseases (NCT/UCC), Dresden, Germany; German Cancer Research Center (DKFZ), Heidelberg, Germany; Faculty of Medicine and University Hospital Carl Gustav Carus, Technische Universität Dresden, Dresden, Germany; Helmholtz-Zentrum Dresden-Rossendorf (HZDR), Dresden, Germany

[3] Department of Neurosurgery, University Hospital Carl Gustav Carus, Dresden, Germany

[4] Department of Anaesthesiology and Critical Care Medicine, Clinical Sensoring and Monitoring, Faculty of Medicine, Technische Universität Dresden, Dresden, Germany

[&] Present address: Department of General, Visceral und Thoracic Surgery, St. Elisabethen-Klinikum Ravensburg, Academic Teaching Hospital of the University of Ulm

SUPPLEMENTARY MATERIAL

[*]Corresponding author:

Dr. med. Christian Teske

Department of Visceral, Thoracic and Vascular Surgery

University Hospital Carl Gustav Carus

Technische Universität Dresden

Fetscherstraße 74

01307 Dresden

Germany

Tel: +49 351 - 458 11909

Email: christian.teske@ukdd.de

Supplementary Figure S1

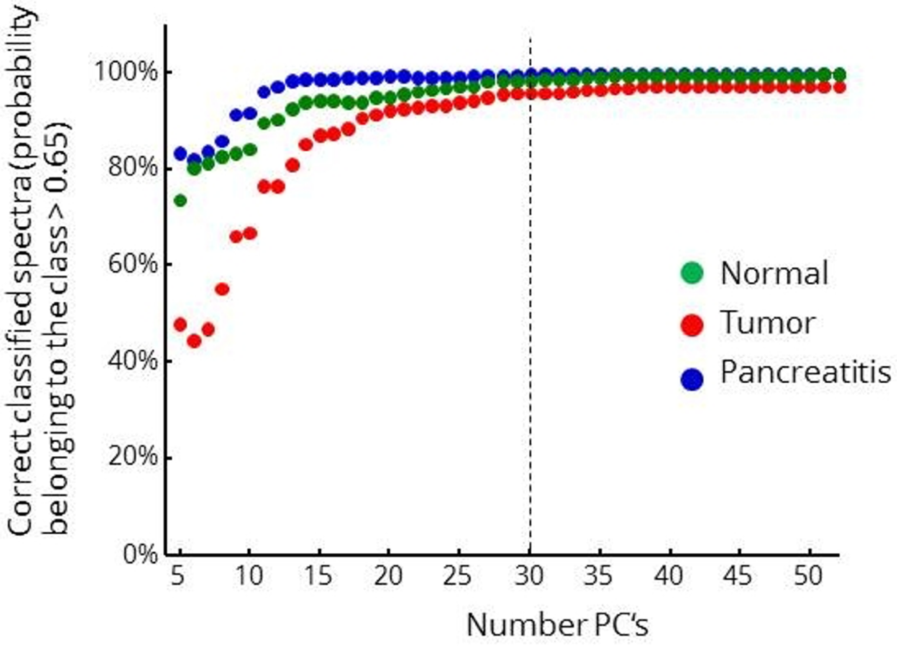

Supplementary Figure S1 Plot accuracy of the classification related to the number of PCs used for the classification algorithm.

Supplementary Figure S2

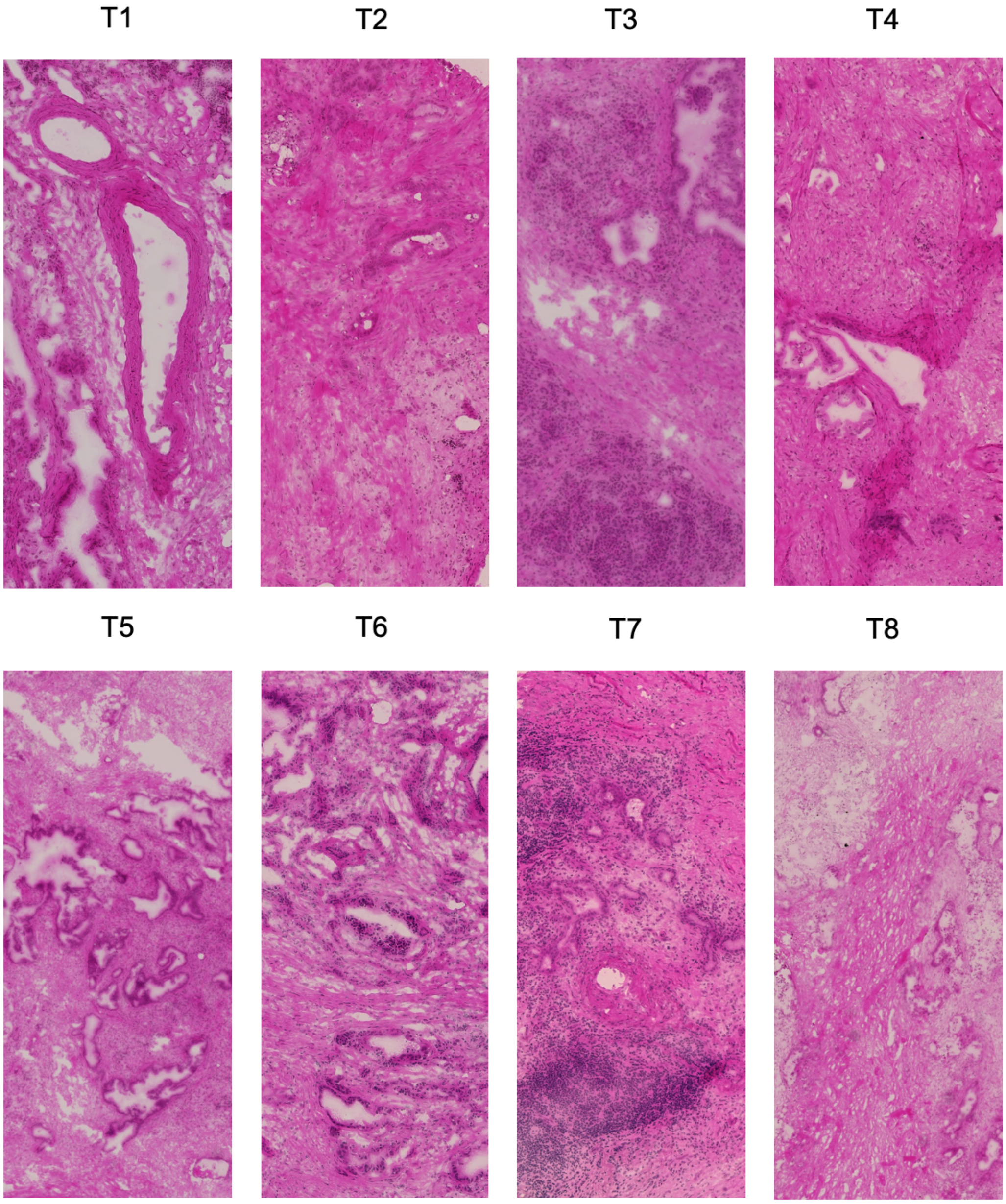

Supplementary Figure S2 Corresponding HE-stained histological images to the PDAC samples used for the classifier.

Supplementary Figure S3

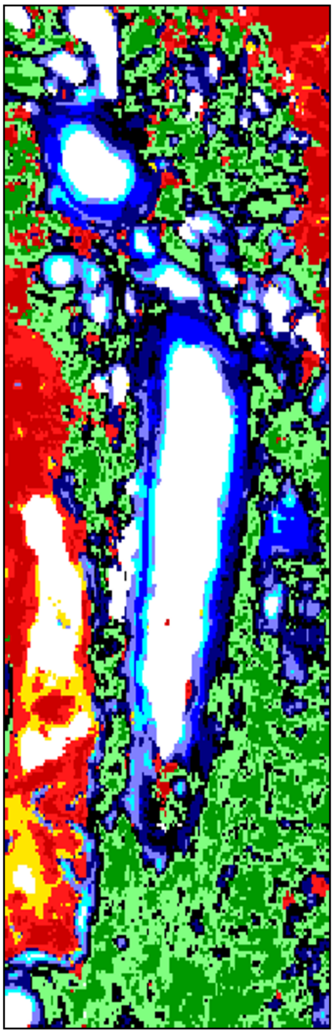

Supplementary Figure S3 Fuzzy k-means cluster analysis was performed with 10 clusters preset by the elbow method. The assignment of red and yellow clusters corresponds well with areas of tumor cells indicated by the histological image. Only spectra belonging to the red and yellow cluster were classified and selected as tumor spectra to build up the training set for supervised classification.
